# Gap Junctional Coupling Between Retinal Astrocytes Exacerbates Neuronal Damage in Ischemia-Reperfusion Injury

**DOI:** 10.1167/iovs.62.14.27

**Published:** 2021-11-30

**Authors:** Abduqodir H. Toychiev, Khulan Batsuuri, Miduturu Srinivas

**Affiliations:** 1 Department of Biological and Vision Sciences, SUNY College of Optometry, New York, NY, United States

**Keywords:** neuroprotection, astrocytes, connexins, retinal ganglion cells, ischemia reperfusion injury

## Abstract

**Purpose:**

Retinal astrocytes abundantly express connexin 43 (Cx43), a transmembrane protein that forms gap junction (GJ) channels and unopposed hemichannels. While it is well established that Cx43 is upregulated in retinal injuries, it is unclear whether astrocytic Cx43 plays a role in retinal ganglion cell (RGC) loss associated with injury. Here, we investigated the effect of astrocyte-specific deletion of Cx43 (Cx43KO) and channel inhibitors on RGC loss in retinal ischemia/reperfusion (I/R) injury and assessed changes in expression and GJ channel and hemichannel function that occur in I/R injury. The effect of Cx43 deletion on neural function in the uninjured retina was also assessed.

**Methods:**

Cx43 expression, astrocyte density and morphology, and RGC death in wild-type and Cx43KO mice after I/R injury were determined using immunohistochemistry and Western blotting. Visual function was assessed using ERG recordings. GJ coupling and hemichannel activity were evaluated using tracer coupling and uptake studies, respectively.

**Results:**

Loss of RGCs in I/R injury was accompanied by an increase of Cx43 expression in astrocytes. Functional studies indicated that I/R injury augmented astrocytic GJ coupling but not Cx43 hemichannel activity. Importantly, deletion of astrocytic Cx43 improved neuronal survival in acute ischemia but did not affect RGC function in the absence of injury. In support, pharmacologic inhibition of GJ coupling provided neuroprotection in I/R injury.

**Conclusions:**

The increase in Cx43 expression and GJ coupling during acute I/R injury exacerbates RGC loss. Inhibition of astrocytic Cx43 channels might represent a useful strategy to promote RGC survival in pathologic conditions.

It is now well established that astrocytes play vital roles in the maintenance of neuronal homeostasis.^1^^,^^2^ In the retina, astrocytes interact closely with retinal ganglion cell (RGC) bodies and axons in the optic nerve head and provide structural and neurotrophic support.^2^ Astrocytes affect RGC function by insulating their axons, clearing neurotransmitters and mitochondrial debris, and buffering extracellular ions.^3^^–^^7^ In response to injury, astrocytes undergo alterations in morphology and gene expression, a process referred to as astrogliosis (or reactivity) that is characterized by an increased expression of glial fibrillary acidic protein (GFAP), hypertrophy, increased production of proinflammatory cytokines, and changes in the expression of extracellular matrix proteins.^5^^,^^7^^–^^10^ These changes are observed in different types of retinal injury, including elevation of IOP, damage to the optic nerve, and after ischemia.^9^^,^^11^^,^^12^ Astroglial reactivity has been shown to both protect and damage neurons, depending on the degree of neuronal injury and the type of insult.^8^^,^^11^^,^^13^^–^^16^

A common feature of astrocytes in the retina and brain is the presence of gap junctions (GJs) that serve to communicate electrical and chemical signals directly between adjacent cells.^17^^,^^18^ GJ channels, comprising proteins called connexins, are formed by the docking of hexameric connexin hemichannels from each adjacent cell. In the retina, GJ coupling in astrocytes is solely provided by connexin 43 (Cx43) and enables these cells to form a functional syncytium.^18^^–^^20^ Astrocytic Cx43 can also form hemichannels in the absence of docking, providing transmembrane and paracrine signaling.^21^^–^^25^ In addition to astrocytes, Cx43 is present in the retinal vasculature and has been detected in Müller glial cell cultures, albeit at much lower expression levels.^26^^–^^28^

The contribution of astrocytic GJ coupling and hemichannels to RGC function and survival is not fully understood. It is established that various types of retinal injury cause an increase in the expression of Cx43 in astrocytes (and endothelial cells)^27^^,^^29^ or an alteration in its phosphorylation status.^18^ These changes have been correlated to changes in the strength of GJ coupling and contribute to either deleterious effects on astrocyte survival by transfer of toxic metabolites in response to hypoxia^18^ or beneficial effects by facilitating metabolite transfer to injured neurons in early glaucomatous injury.^30^ Pharmacologic studies also implicate aberrant hemichannel activation in disease progression, although studies indicate an involvement of Cx43 hemichannels in endothelial cells rather than astrocytes.^27^^,^^31^

In this study, we examined the role of astrocytic Cx43 on RGC survival in a mouse ischemia/reperfusion (I/R) model. We show that I/R injury produces a loss of RGCs that is paralleled by upregulation of astrocytic Cx43, an increase of astrocytic GJ coupling but not hemichannel opening, and astrocytic hypertrophy. Genetic ablation of astrocytic Cx43 or inhibition of GJ coupling significantly improves RGC survival. In addition, we find that Cx43 deletion does not alter retinal function in the absence of injury as assessed by ERG recordings, indicating that Cx43 might represent a viable target for neuroprotection in the retina.

## Methods

### Animals

All animal procedures were conducted in accordance with ARVO statement regarding use of animals in vision research and were approved by the State University of New York Optometry Institutional Animal Care and Use Committee. Experiments were performed on retinas obtained from C57/BL6, conditional Cx43KO, and GFAPtd mice. All animals purchased from Jackson Laboratories and animals of both sexes were used for experiments. Astrocyte-specific Cx43 conditional knockout mice were generated with the Cre/LoxP system by breeding floxed Cx43 mice (stock no. 008039) to mouse glial fibrillary acidic protein mGFAP-Cre mice (stock no. 012886), as previously reported.^18^ Astrocytes labeled with td-Tomato were created by crossing loxP-tdTomato Ai9 reporter mice (stock no. 007909) with mGFAP-Cre mice.

### I/R Injury Model

I/R injury was induced as previously described, with minor modifications.^32^ Briefly, 2-month-old mice were anesthetized by intraperitoneal injections of ketamine 70 mg/kg and xylazine 7 mg/kg. The pupil was dilated with 1% tropicamide (Henry Schein, Inc., Melville, NY, USA), and cornea was topically anesthetized with 0.5% proparacaine hydrochloride (Alcon, Fort Worth, TX, USA). The anterior chamber of one eye was cannulated with a 30-gauge needle connected to a reservoir of sterile saline. The reservoir was raised to produce an IOP of 75 to 80 mm Hg for 60 minutes. Ischemia was confirmed by the whitening of the eye. After 60 minutes, the needle was withdrawn, and reperfusion was confirmed by the reappearance of normal black color to the iris. The contralateral sham-treated eye was used as control. Temperature was maintained using a heat pad. IOP was measured using a tonometer (TonoLab, Franconia, NH, USA). An antibacterial ointment, vetropolycin, was applied to prevent infection. Enucleated eyes after euthanization were processed after 1, 3, or 7 days postinjury (dpi).

### Intracellular Neurobiotin Injections

Experimental procedures are similar to our previously reported work.^18^ Briefly, eyes were enucleated and retinas were dissected in oxygenated HEPES buffer, transferred to filter papers (12-mm diameter cell culture inserts with 0.4-µm pore size; Millipore Sigma, Burlington, VT, USA), and kept in a chamber perfused with oxygenated bicarbonate extracellular buffer. A single tdTomato-labeled astrocyte was injected with the GJ permeable tracer Neurobiotin (NB; 5 mg/mL) using a 15-mV positive current for 15 minutes in the whole-cell configuration. NB-filled cells were visualized using Alexa Fluor 488–conjugated streptavidin, which binds to NB. To ensure that the injected cells are astrocytes, retinas were stained with a mouse anti-GFAP primary antibody followed by donkey anti-mouse secondary antibody conjugated with Alexa 674. Astrocytes labeled with NB were counted manually in images obtained with confocal microscopy using ImageJ software (National Institutes of Health, Bethesda, MD, USA).

### EtBr Uptake Assay

Ethidium bromide (EtBr) uptake assay was performed as previously described.^18^ Briefly, eyes were enucleated, and retinas were dissected and incubated in PBS supplemented with 2 mM CaCl_2_ and 1 mM MgCl_2_ in the presence or absence of 300 µM Gap19 (Millipore Sigma) for 15 minutes at room temperature. Then, 4 µM EtBr (Millipore Sigma) was added to the solution for 10 minutes. Retinas were rinsed with PBS, fixed with paraformaldehyde (PFA) for 30 minutes, and stained against GFAP for confocal imaging. Uptake of EtBr was assessed by quantification of relative fluorescence intensity of EtBr in astrocytes. Relative fluorescence intensity was obtained by subtracting the mean fluorescent intensity of astrocytes from background fluorescence.

### Intravitreal Injections

Mice were anesthetized with intraperitoneal injections of a mixture of ketamine 70 mg/kg and xylazine 7 mg/kg. The Cx43 inhibitor SBO15 (compound 18 = 5-(4-phenoxybutoxy) isoquinoline) (see Ref^19^) was diluted in PBS and injected intravitreally at a concentration of 20 µM to the left eyes one day after I/R. The right eyes received PBS to serve as control (sham).

### Immunohistochemistry

For whole-mount preparations, eyes were enucleated and retinas were isolated and fixed in PFA solution (4% in PBS; Santa Cruz Biotechnology, Dallas, TX, USA) for 15 minutes at room temperature. Tissue was incubated with blocking buffer containing 5% Chemiblocker (Millipore Sigma, Burlington, VT, USA), 0.5% Triton X-100, and 0.05% sodium azide (Millipore Sigma) for 1 hour. Whole mounts and vertical frozen sections were incubated with appropriate primary antibodies overnight at 4 °C, washed with PBS, and further incubated with fluorescent secondary antibodies for 1 hour at room temperature. Primary antibodies included goat anti-Brn3a (1:1000; Santa Cruz Biotechnology), mouse anti-GFAP (1:1000; Thermo Fisher Scientific, Waltham, MA, USA), rabbit anti-Cx43 (1:1000; Millipore Sigma), and rabbit anti-Sox9 (1:500; Millipore Sigma). Primary antibodies were diluted in the same blocking buffer and applied for 24 hours, followed by a 3-hour incubation with appropriate secondary antibody, Alexa 488 (1:500; Thermo Fisher Scientific), Alexa 594 (1:500; Thermo Fisher Scientific), and Alexa 647 (1:500; Thermo Fisher Scientific). Imaging was performed using a confocal microscope (Olympus, Tokyo, Japan) and processed and analyzed with ImageJ software (National Institutes of Health). The cell counter function in ImageJ was used to measure astrocyte and ganglion cell numbers. Cx43 puncta were analyzed with puncta analyzer (ImageJ plug-in), with the threshold for pixel size set at 1 to 20. GFAP mean fluorescence pixels were measured in a selected area of 0.1 mm^2^. Density analysis was performed to assess GFAP-positive astrocytic processes using the particle analysis function in ImageJ. Images were converted to a black and white image with the threshold set above 4; this threshold value reduced interference from activated Müller cell end-feet from density analysis of astrocyte reactivity in injury. The threshold parameters were identical for each measurement when comparing different groups. Quantification of GFAP-positive astrocyte reactivity was measured as the percentage of area occupied by GFAP labeling in each region of interest.

### Western Blot of Cx43

Western blot was performed as previously described.^18^ Briefly, four retinas from naive or injured mouse eye were dissected in ice-cold PBS and then lysed in 250 µL RIPA buffer (Millipore Sigma) supplemented with 1% protease inhibitor mixture (Millipore Sigma) and 1× phosphatase inhibitor mixture (Roche). The protein concentration was quantified using a BCA protein assay kit (Thermo Fisher Scientific); then, 20-µg of protein was loaded and separated by 12% polyacrylamide gel electrophoresis and transferred to polyvinylidene difluoride membranes. After blocking with 1.5% ECL prime (Cytiva Amersham, Marlborough, MA) blocking solution in tris buffered saline, 0.1% Tween 20 (TBST) for 1 hour, the blots were immunolabeled with primary antibodies against rabbit anti-Cx43 (1:6000; Millipore Sigma) or mouse anti-tubulin (1:20,000; Millipore Sigma) overnight at 4°C. Then, blots were washed with TBST and incubated with horseradish peroxidase–conjugated secondary antibody goat IgG (1:20,000; Santa Cruz) at room temperature for 1 hour. The membrane blots were washed and visualized by SuperSignal West Femto substrate (Thermo Fisher Scientific). The intensity of the band was imaged with the iBright imaging system (Thermo Fisher Scientific) and quantified by densitometry using ImageJ software.

### ERG Recordings

Scotopic ERGs were recorded as described previously.^18^ Briefly, 3- to 5-month-old mice were dark adapted for 18 to 24 hours. Animals were anesthetized with intraperitoneal administration of 70 µg ketamine/g body weight and 7 µg xylazine/g body weight diluted in saline. Pupils were dilated with topical tropicamide solution (1%) (Henry Schein, Inc.). Platinum electrodes were placed at the cornea of both eyes. The reference electrode was inserted into the cheek and the ground electrode was inserted under the back skin. ERG responses to brief white LED test flashes with intensities in the range of −6.7 to 2.9 log candela (cd)⋅s/m^2^ for positive scotopic threshold response (pSTR) and −3.0 to 0.3 log cd⋅s/m^2^ for a- and b-waves were recorded using the Espion electrodiagnostic system (Diagnosys LLC, Lowell, MA, USA). Responses of both eyes to each stimulus intensity were averaged to represent a single data point. The data value represents the maximum response amplitude of a-wave, b-wave, and pSTR.

### Statistics

Statistical analyses were performed using the Origin7pro software, Northampton, MA, USA. Results are presented as mean ± SD. *n* values correspond to the number of mice used in ERG experiments and *n* values correspond to the number of retinas used in all other experiments. One-way ANOVA and unpaired two-sample Student's *t*-test at the significance level of α = 0.05 (95% confidence interval) was used for comparisons between two mice groups of different genotypes.

## Results

### Effect of Cx43 Deletion on Normal Retinal Function

**Figure 1.**
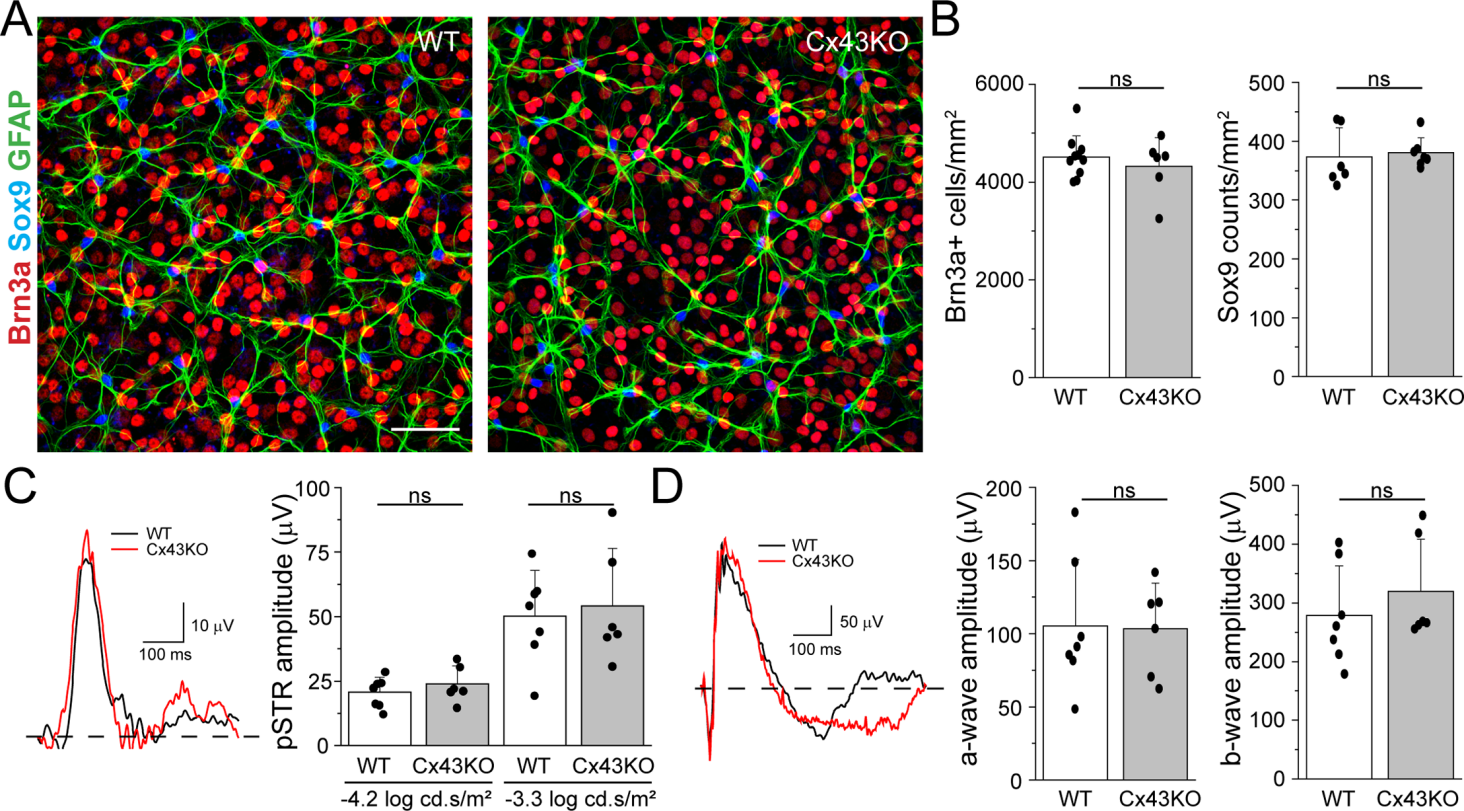
Effect of Cx43 deletion in astrocytes on normal retinal function. (**A**) Representative confocal images from WT (*left panel*) and Cx43KO (*right panel*) mouse retinas immunostained with Brn3a (*red*), GFAP (*green*), and Sox9 (*blue*). *Scale bar*: 50 µm. (**B**) Quantification of Brn3a-positive RGCs (*left*) and Sox9-positive (*right*) cells in WT and Cx43KO mice (*n* = 6–9). Data show no significant difference in the number of Brn3a- and Sox9-positive cells. (**C**) pSTR recorded at −3.3 log cd⋅s/m² from WT (*n* = 7) and Cx43KO (*n* = 6) mice. *Dashed line* indicates baseline. The amplitudes of pSTR at two different intensities did not show significant differences between WT and Cx43KO. (**D**) Representative ERG traces recorded at −0.3 log cd⋅s/m² and quantification of the a-wave (*left*) and b-wave (*right*) amplitude. Data are presented as mean ± SD. Student's *t*-test, not significant (ns) *P* > 0.05.

In a previous study, we showed that deletion of Cx43 in astrocytes markedly reduced Cx43 labeling in astrocytes and reduced retinal expression of Cx43 protein by ∼90%.^18^ Functional studies further showed that absence of Cx43 completely reduced GJ coupling as assessed by NB transfer,^18^ indicating that other connexin subtypes are not expressed in astrocytes or upregulated after Cx43 deletion. The inability of astrocytes to form a coupled network in Cx43 KO retinas did not affect angiogenesis during early postnatal development and did not alter the structure and function of the neural retina.^18^ The thickness of the outer and inner nuclear layers was not different between wild-type (WT) and Cx43 KO mice. Consistent with these histologic findings, ERGs showed that scotopic a- and b-wave amplitudes of Cx43 KO retinas were not significantly different from WT retinas at P28. To further characterize whether Cx43 deletion affects normal retinal function in adult animals (3–5 months of age), we measured the numbers of RGCs and astrocytes in Cx43 flox (WT) and knockout (KO) littermates, using Brn3a and Sox9 labeling, respectively (Figs. 1A, 1B). There was no difference in Brn3a^+^ RGC and astrocyte numbers between WT and KO mice. In addition, the amplitude of pSTR, a measure of RGC function, was also measured at two different intensities (Fig. 1C). There was no significant difference in pSTR amplitudes between WT and Cx43 KO mice (Fig. 1C). There was a small but significant change in the latency to the peak of the pSTR at the lower intensity (131.5 ± 8.17 ms and 120.4 ± 3.85 ms for WT and Cx43KO, respectively, at −4.2 log cd⋅s/m^2^; *P* = 0.024), but this effect was not seen at the higher light intensity (*P* = 0.89) (data not shown). Finally, scotopic a- and b-wave amplitudes were also unaffected by Cx43 deletion in astrocytes in adult mice (Fig. 1D). Taken together, these results indicate that astrocyte Cx43 does not significantly affect normal RGC function.

### Ischemic Reperfusion Injury Induces RGC Death but Does Not Affect Astrocyte Numbers

**Figure 2.**
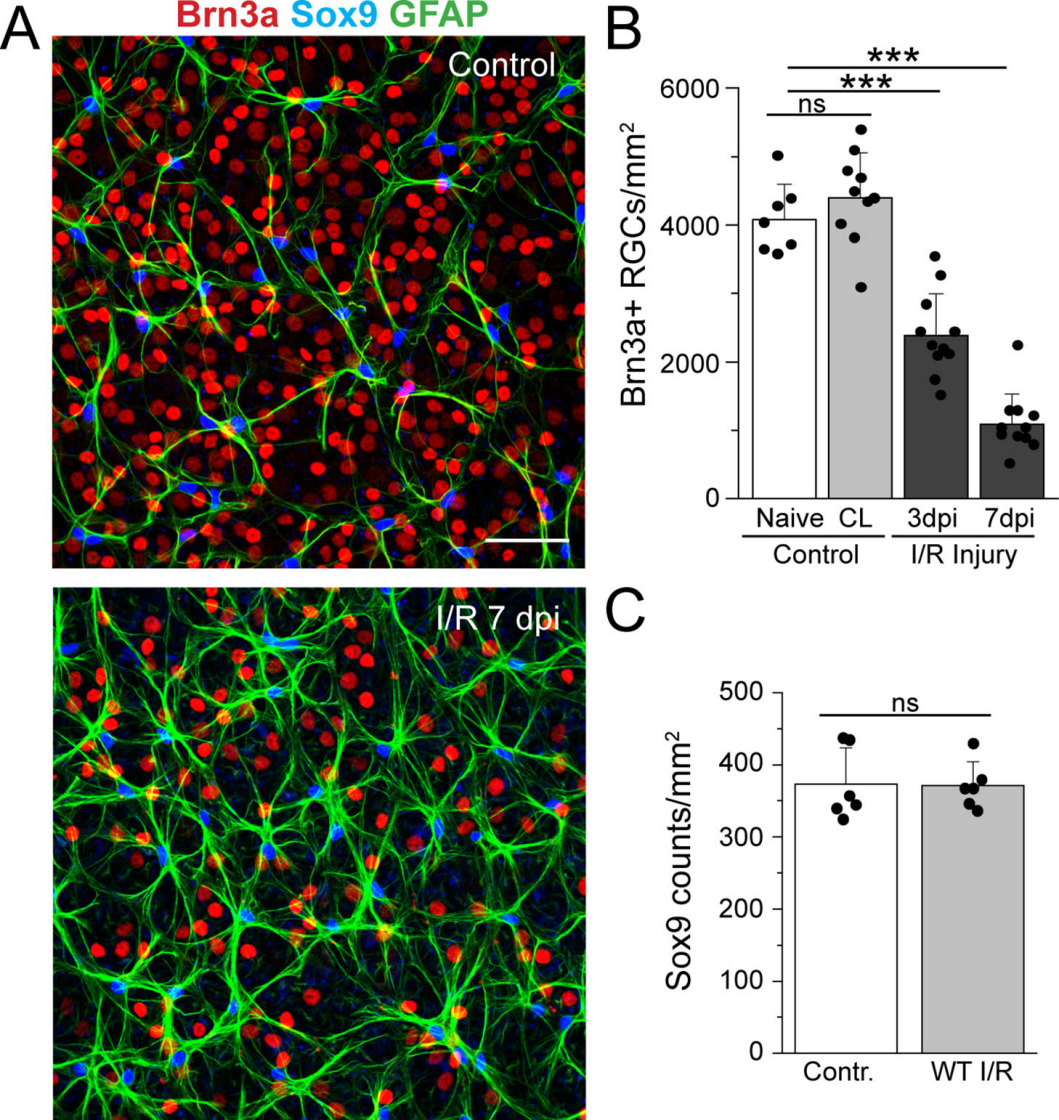
Loss of RGCs in retinal ischemia reperfusion model. (**A**) Representative confocal images from control (*top panel*) and 7 days after I/R injury (*bottom panel*) acquired from whole-mount staining of WT mouse retinas labeled with Brn3a (*red*), GFAP (*green*), and Sox9 (*blue*). *Scale bar*: 50 µm. (**B**) Quantification of the number of Brn3a-positive cells for naive (*n* = 7), contralateral (CL) (*n* = 10), and I/R retinas at 3 (*n* = 11) and 7 (*n* = 11) dpi. There was a significant reduction of RGCs at 3 and 7 dpi control. (C) Quantification of Sox9-positive cells in the nerve fiber layer (NFL). Data show no significant change in number of astrocytes after 7 dpi I/R injury, *P* = 0.96 versus control. Data are presented as mean ± SD. One-way ANOVA is used in **B**, ns *P* > 0.05; ***P < 0.001 versus control. Student's *t*-test is used in **C**, ns *P* > 0.05.

I/R injury was induced by elevating intraocular pressure as described previously (see Methods). This procedure caused a significant increase in RGC death.^32^^–^^35^ To evaluate RGC loss, retinal whole mounts were labeled with the selective RGC marker Brn3a, which labels 85% to 90% of RGCs in the rodent retina.^36^ Cell counts were obtained from midperipheral regions from the four retinal quadrants of identical size (∼1.5–2.0 mm from the optic disk). Brn3a labeling in retinas after I/R injury was compared with contralateral and naive retinas in WT mice. As shown in Figure 2, Brn3a^+^ RGC numbers, assessed at 3 and 7 dpi, decreased by 43.4% ± 4.3% and 74.1% ± 3.1%, respectively, compared with naive control eyes (*P* < 0.001). In addition, we found that Brn3a^+^ RGC numbers in contralateral retinas were not different from those obtained from naive eyes (Fig. 2B).

In contrast, I/R injury did not cause a significant change in the density of astrocytes. Astrocyte numbers were assessed using Sox9, which labels nuclei of glial cells in the retina and the brain.^37^ No significant differences in the densities of astrocytes were observed between control and 7 days after I/R injury. However, I/R injury caused marked morphologic changes in astrocytes, including hypertrophy of the astrocytic cell soma and processes, consistent with reactive remodeling (see below).

### Retinal I/R Injury Upregulates Cx43 in Astrocytes and Promotes Gliosis

**Figure 3.**
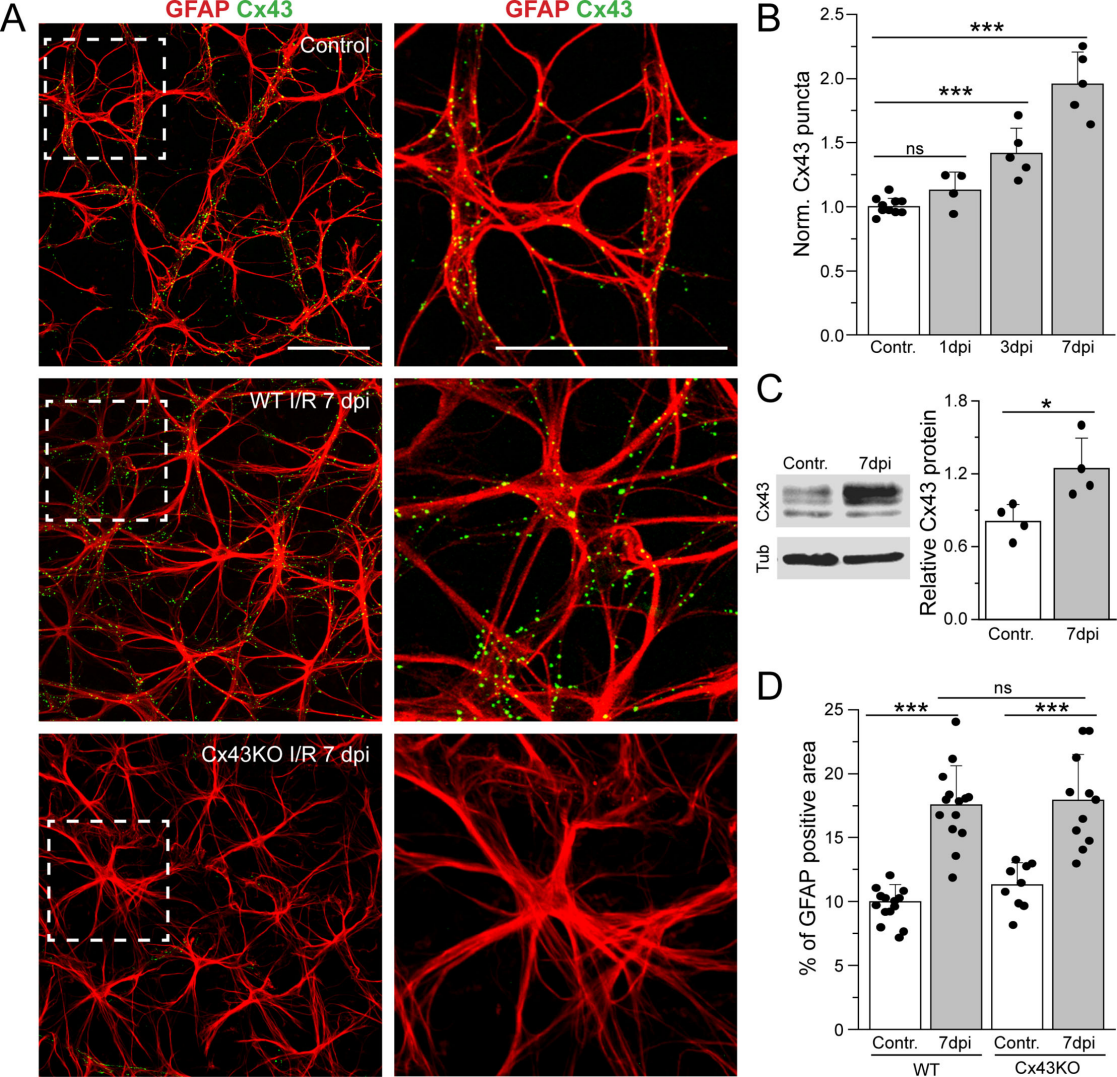
Cx43 expression is upregulated in reactive astrocytes in I/R injury. (**A**) Representative images from the NFL that shows expression of gap junction protein Cx43 (*green*) in astrocytes stained with GFAP (*red*) in control and I/R retinas from WT and Cx43KO mice. Panels on the *left* are magnified images from the insert (*white box*). *Scale bar*: 50 µm. (**B**) Normalized Cx43 puncta per area shows an increase of Cx43 expression in I/R retina at 1 dpi (*n* = 4), 3 dpi (*n* = 5), and 7 dpi (*n* = 5) compared with control (*n* = 10). (**C**) Western blot of retinal lysates of Cx43 in control and after I/R injury (*left*). Quantification (*right*) of total Cx43 protein in WT retina showing a significant increase in Cx43 protein expression after 7 dpi I/R injury (*n* = 4). (**C**) Quantification of the astrocytic density based on GFAP staining (*red*) of WT (*n* = 14–15) and Cx43KO (*n* = 10–12) retinas in control and I/R injury. GFAP-positive areas were increased in both WT and Cx43KO mice after 7 dpi I/R injury. Data are presented as mean ± SD. Student's *t*-test, ns *P* > 0.05; *P < 0.05; ***P < 0.001 versus control.

Upregulation of Cx43 and gliosis has been observed in various retinal diseases and in various diseases models, including the rat I/R injury model.^29^ Changes in expression of Cx43 and astrocyte reactivity in I/R injury also occur in mice. Figure 3 shows immunolabeling for Cx43 in control and after I/R injury from WT and Cx43KO mice. Consistent with previous studies,^18^^,^^38^ intense punctate staining of Cx43 in GFAP-expressing astrocytes can be observed in retinal whole-mount preparations in control. I/R injury induced a significant increase in Cx43 labeling in astrocytes (Fig. 3A). The increase in the number of Cx43 puncta in astrocytes in WT retinas occurred as early as 1 dpi after I/R injury and showed a gradual increase at 3 dpi and 7 dpi of I/R injury compared with contralateral control eyes (Fig. 3B). In comparison, Cx43 puncta were virtually absent in retinas in which Cx43 was genetically deleted from GFAP-expressing cells (Fig. 3A).

The increase in Cx43 immunolabeling was confirmed by Western blotting of lysates obtained from control and I/R retinas (Fig. 3C). Cx43 protein exhibits multiple differentially migrating isoforms when analyzed by electrophoresis.^39^^–^^41^ A faster migrating band that includes the nonphosphorylated isoform (referred to as the P0 band) is observed at 40 kDa, and at least two slower migrating forms (referred to as P1 and P2) are seen at 41 kDa and 43 kDa, respectively. In response to I/R injury, there was a large increase in both the P1 and P2 forms, whereas the P0 form of Cx43 was unaffected. The total Cx43 protein was obtained by combining the bands corresponding to the three distinct isoforms and indicated that Cx43 protein levels were significantly upregulated after I/R injury (Fig. 3C).

We also examined whether the elimination of astrocytic Cx43 alters the morphologic changes to astrocytes associated with reactive remodeling. Therefore, we performed density analysis of astrocytes in whole-mount retina from WT and Cx43KO mice in control and after I/R injury (Fig. 3D). There was an increase in GFAP-positive processes in astrocytes at 3 dpi; this density was even higher at 7 dpi compared with control (*P* < 0.0001). Density analysis of reactive astrocytes in Cx43KO showed that the Cx43 does not affect reactive remodeling changes in astrocyte processes.

### Astrocytic Gap Junctional Coupling Is Elevated in I/R Injury

**Figure 4.**
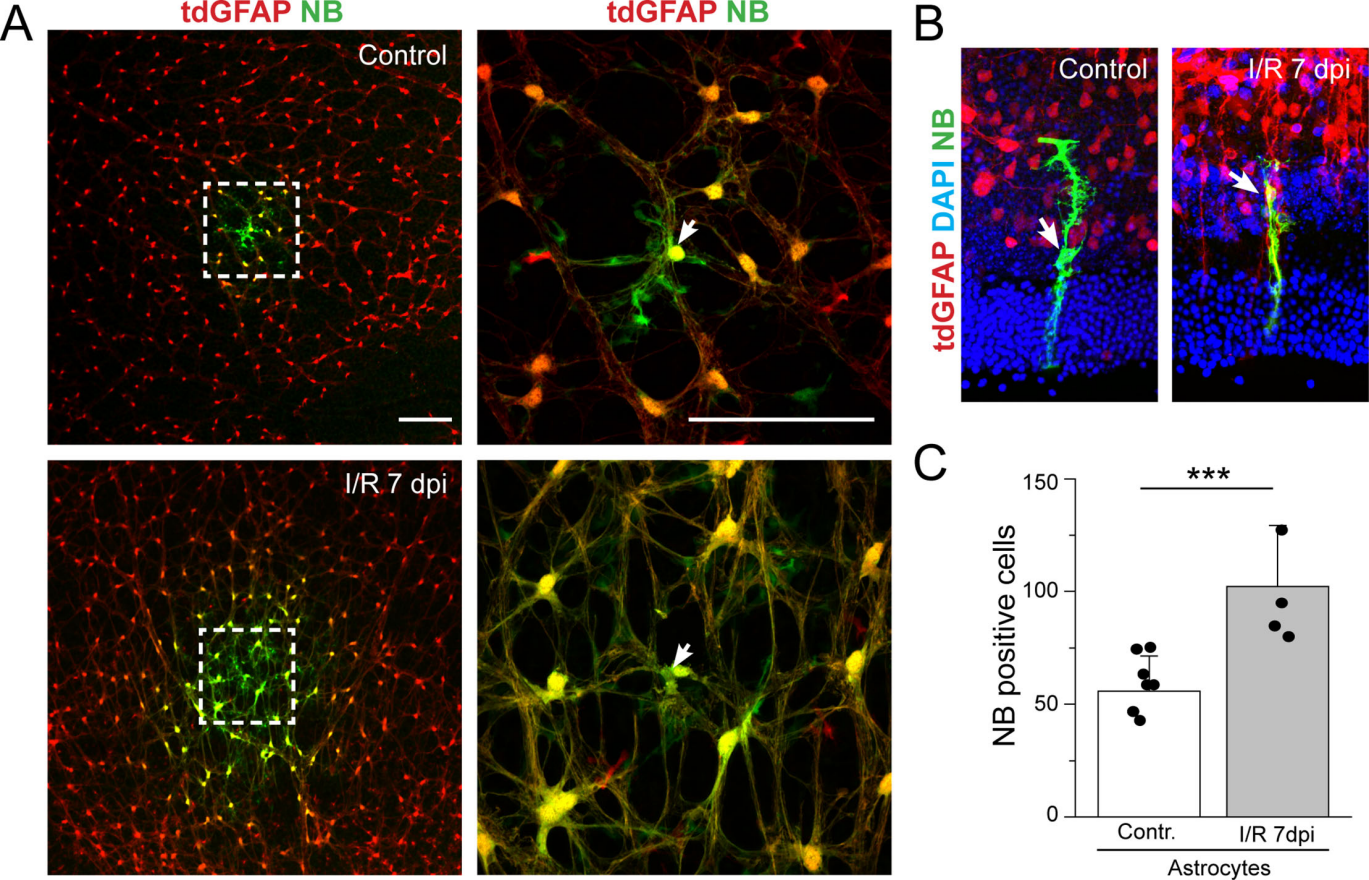
I/R injury increases GJ coupling in astrocytes but not in Müller cells. (**A**) Representative confocal image of astrocytes expressing tdTomato (*red*) showing diffusion of the tracer NB between coupled astrocytes in control and I/R injury retinas. The panel on the *right* shows a magnified image from the *left panel* and indicating the NB-loaded cell (*white arrow*). *Scale bar*: 50 µm. Coupled astrocytes were visualized with streptavidin 488 (*green*) after loading single astrocytes with NB for astrocytes. (**B**) Representative confocal images of Müller cells injected with NB (*green*) in control (*n* = 5) and after 7 dpi I/R injury (*n* = 4) showing no coupling between Müller cells. Cell nuclei were stained with DAPI (*blue*). (**C**) Quantification of the NB-positive cells at control (*n* = 6) and I/R injury at 7 dpi (*n* = 4). Data are presented as mean ± SD. Student's *t*-test ***P < 0.001 versus control.

To examine whether the upregulation in the expression of Cx43 by I/R injury leads to an augmentation of GJ connectivity, we introduced NB into individual astrocytes in retinal whole mounts using whole-cell patch clamp. To facilitate identification of astrocytes, we used a reporter mouse line expressing tdTomato in astrocytes (by crossing a tdTomato reporter mouse with a mouse expressing cre recombinase in GFAP locus). After loading an individual cell for 15 minutes, the retinas were fixed and then labeled with streptavidin conjugated to Alexa 488. In uninjured retina, injection of the NB into an individual astrocyte resulted in the diffusion of the dye to multiple adjacent astrocytes (Fig. 4A). I/R caused a marked increase in the strength of astrocytic GJ coupling. The increase in GJ coupling (approximately twofold) was detected within 24 hours of ischemic injury and persisted for up to 7 dpi (Fig. 4C).

We also labeled retinal whole mounts with markers for RGCs (Brn3a) and microglia (Iba1) and did not find colocalization of streptavidin-488 with RGCs or microglia (data not shown), indicating that astrocytes do not couple with these cell types in the retina. Expression of Cx43 and low levels of GJ coupling has been documented between Müller cells in some species such as rabbit.^42^^,^^43^ However, Müller glia in rat retinas showed little evidence of GJ coupling, as assessed by tracer spread.^44^ In order to clarify this discrepancy, we injected NB into Müller cells before and after I/R injury. Our data show no transfer of the tracer to adjacent Müller cells in control or after I/R injury (Fig. 4B), consistent with previous studies.^19^^,^^44^

### I/R Injury Does Not Significantly Increase Hemichannel Activity

**Figure 5.**
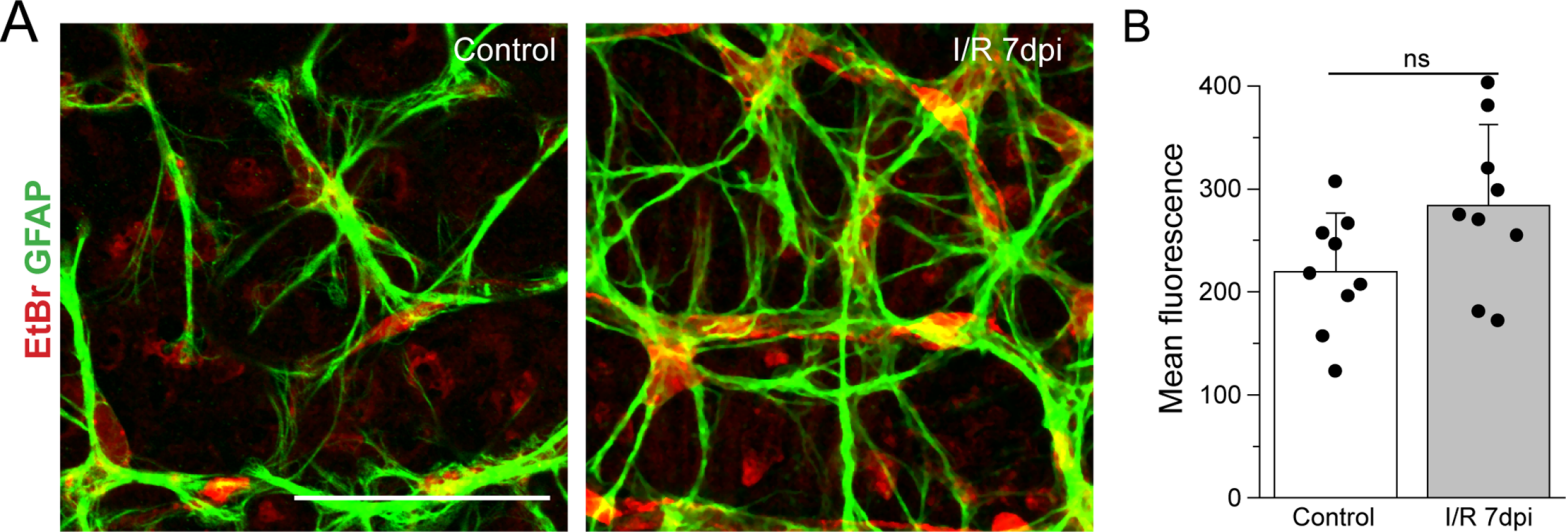
Cx43 hemichannel activity is not in I/R injury. Hemichannel activity in astrocytes was measured by EtBr uptake. (**A**) Images of whole-mount retinas showing EtBr uptake (*red*) labeled with anti-GFAP (*green*) in WT control (*left panel*) and at 7 dpi (*right panel*). *Scale bar*: 50 µm. (**B**) Relative fluorescent intensity of astrocytes shows difference in EtBr uptake between control (*n* = 9) and I/R (*n* = 9) retinas. Data are presented as mean ± SD. Student's *t*-test, ns *P* > 0.05.

Previous studies suggest that opening of astrocytic Cx43 hemichannels leads to excessive release of glutamate and ATP and causes neuronal death in ischemic injury in the brain.^21^^,^^45^^–^^47^ To evaluate whether Cx43 hemichannel activity is augmented in retinal I/R injury, we measured connexin-specific EtBr dye uptake as previously described.^18^ EtBr uptake assay was performed in PBS solution supplemented with divalent cations. We found that uptake of EtBr by astrocytes was slightly increased in both control and I/R retina after 7 dpi (Fig. 5). The difference in intensity of dye uptake between control and I/R retina was insignificant in astrocytes and not blocked by Gap19, a peptide that specifically blocks Cx43 hemichannels. These results suggest that Cx43 hemichannels do not mediate neuronal damage in the retinal I/R model.

### Genetic Ablation of Astrocytic Cx43 or Pharmacologic Inhibition of Cx43 Channel Improves Survival of RGCs

**Figure 6.**
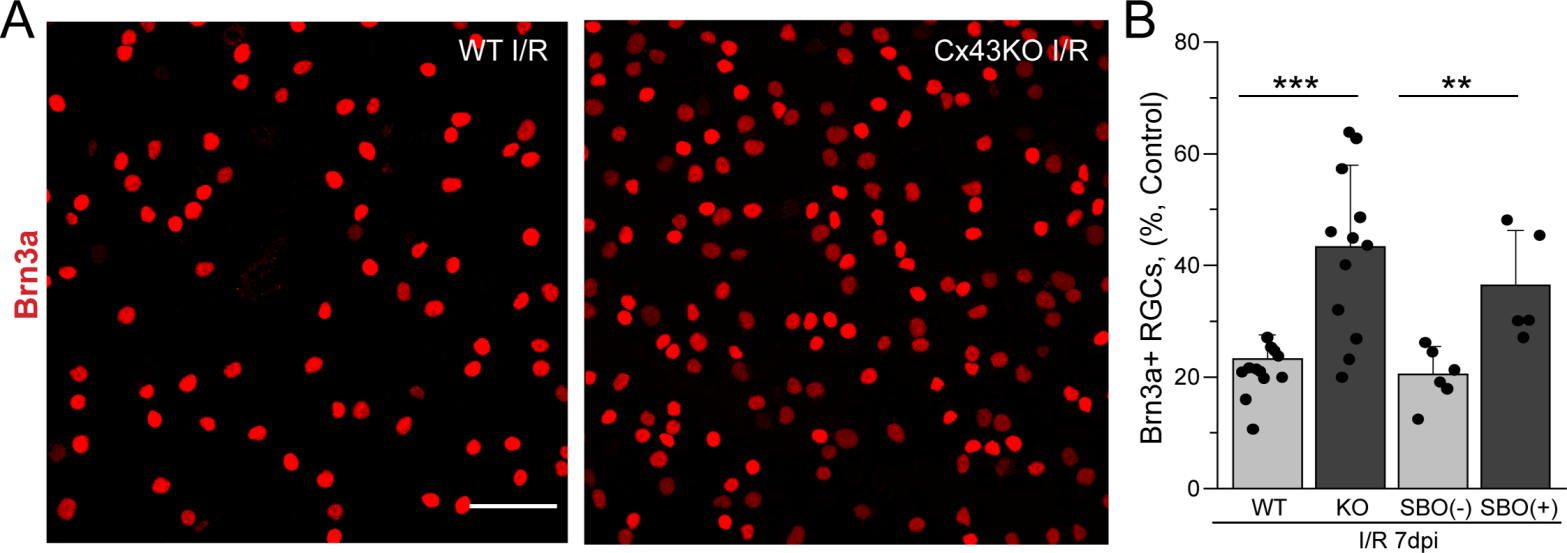
Effect of conditional deletion of astrocytic Cx43 and pharmacologic inhibition of Cx43 channels on RGC survival in I/R injury. (**A**) Representative images of I/R retinas from WT (*left panel*) and astrocyte-specific Cx43KO (*right panel*) immunostained against Brn3a (*red*) in whole-mount retina. (**B**) Quantification of Brn3a-positive cells (per 1 mm^2^) in WT (*n* = 15) and KO (*n* = 10) retinas and in those intravitreally injected with SBO15, an inhibitor of Cx43 channels, 7 days postinjury. Values were normalized to contralateral untreated eyes and depicted as a percentage of RGC survival. Data are presented as mean ± SD. Student's *t*-test ***P < 0.001; **P < 0.01 versus WT I/R.

To determine whether the extensive GJ coupling affects neuronal survival under pathologic conditions, we compared RGC loss in Cx43KO and WT retinas after I/R injury. Brn3a labeling indicated a 2.5-fold increase in RGCs in Cx43 KO retinas compared with WT after 7 days following I/R injury (*P < 0.001), indicating that Cx43 deletion is neuroprotective in I/R injury (Fig. 6). To confirm that this protection is due to Cx43 channel activity, we injected a specific Cx43 channel inhibitor, SBO15 (20 µM), intravitreally 24 hours after I/R injury. In a previous study, we showed that SBO15 selectively inhibits Cx43 channels without affecting channels formed by other connexin subtypes present in the retina.^18^ SBO15 also reduced astrocyte degeneration in vivo in a mouse model of oxygen-induced retinopathy.^18^ Intravitreal injection of SBO15 caused a significant increase in Brn3a^+^ RGC viability following 7 days of I/R injury (*P* < 0.01), **P < 0.01 versus I/R (–) SBO15 (Fig. 6). Collectively, these experiments indicate that elimination of astrocytic GJ coupling improves Brn3a^+^ RGCs survival after I/R injury.

**Figure 7.**
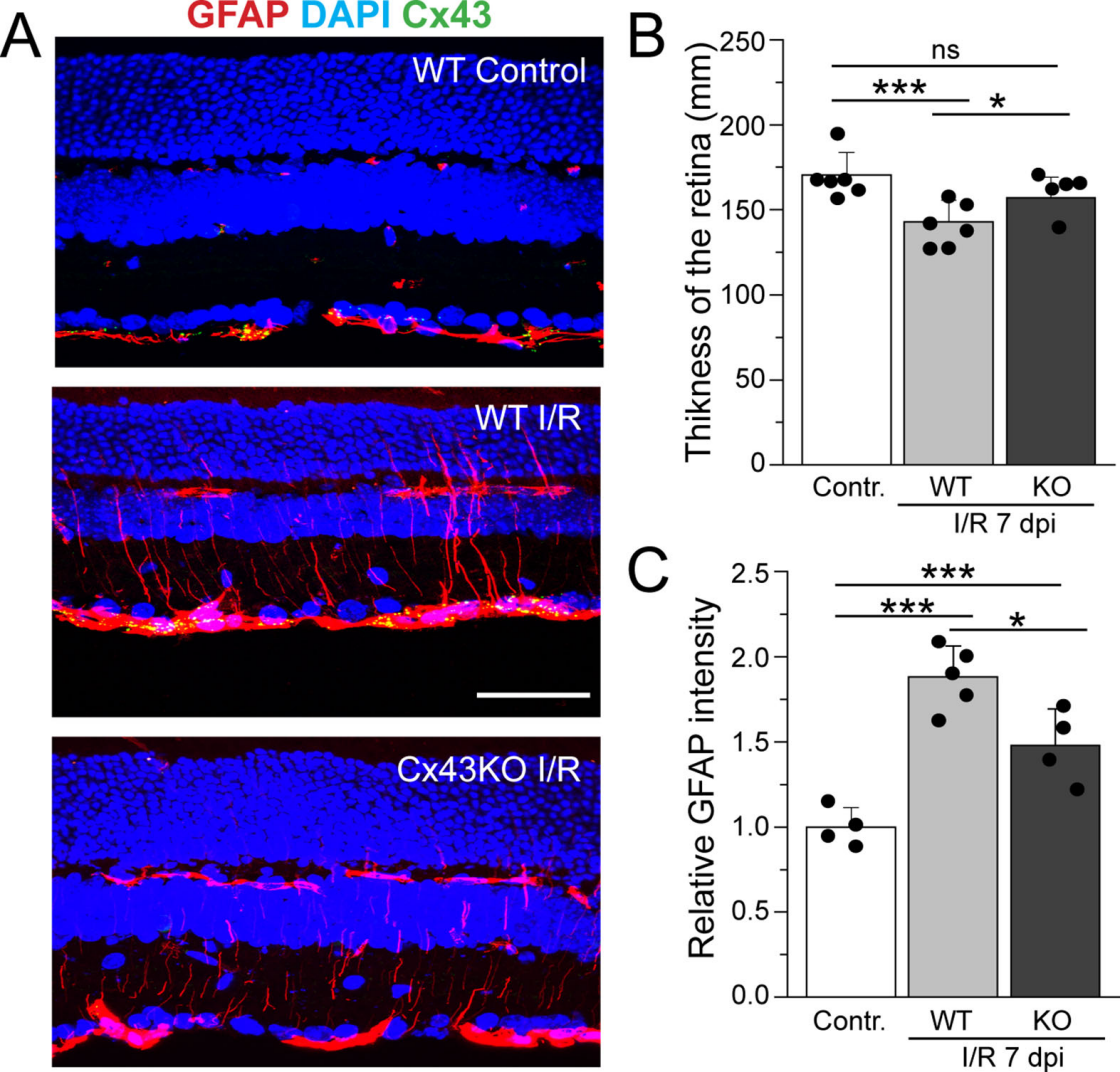
Changes in retinal thickness and GFAP expression in WT and Cx43KO retinas in control and I/R injury. (**A**) Representative images of vertical sections obtained from WT and Cx43KO retinas stained with DAPI (*blue*) and GFAP (*red*). Images demonstrate the retinal thinning and GFAP elevation in control and I/R retinas at 7 dpi. *Scale bar*: 50 µm. (B) Quantification of the total thickness of the retina in WT control (*n* = 5) and after I/R injury in WT (*n* = 6) I/R injured retina. The thickness in Cx43KO (*n* = 5) was insignificant compared with control. *P* = 0.3 versus control. (**C**) Quantification of GFAP immunolabel in WT control (*n* = 4), WT I/R (*n* = 5), and Cx43KO I/R (*n* = 4) retinas. GFAP level is measured as mean fluorescent pixels in a 0.1-mm^2^ area. GFAP intensity shows a significant increase in I/R injury in WT and Cx43KO mice compared with WT control. GFAP intensity was significantly lower in Cx43KO I/R retinas when compared with WT I/R *P* < 0.018. Data are presented as mean ± SD. One-way ANOVA is used in **B** (ns *P* > 0.05; *P < 0.05 versus WT I/R; ***P < 0.001 versus control) and in **C** (*P < 0.05 versus WT I/R; ***P < 0.001 versus control).

I/R injury reduces retinal thickness due to loss of neuronal cells^48^ and increases GFAP expression in Müller glia. To determine whether the increases in Brn3a^+^ RGC survival caused by astrocytic Cx43 ablation have downstream effects on retinal thickness and GFAP expression, we stained vertical sections of WT control, WT I/R, and Cx43KO I/R retinas with the nuclear marker DAPI and GFAP. DAPI labeling revealed a significant decrease in thickness of the WT retina after 7 dpi compared with control (Fig. 7B). The decrease in retinal thickness was mainly due to thinning of the inner plexiform layer (data not shown). This decrease in retinal thickness exhibited a partial recovery in Cx43KO (Figs. 7A, 7B). We also found that deletion of Cx43 reduced GFAP expression in the retina. In control retinas, GFAP expression was localized exclusively in astrocytes in the ganglion cell layer (GCL). In I/R injury, consistent with reactivity, expression of GFAP was significantly upregulated in Müller cell processes in WT retinas but showed only a modest increase in Cx43KO retinas (Fig. 7C). This partial recovery of GFAP upregulation seen in Cx43KO retinas is likely due to neuroprotection afforded by Cx43 deletion rather than a direct effect on Müller cells, because these glial cells were not found to be coupled to each other by GJs (see Fig. 4B).

## Discussion

Astrocytes are spatially distributed in the nerve fiber layer in the retina and function as a syncytium by virtue of their extensive GJ coupling. The function of this widespread astrocytic coupling in retinal physiology and pathophysiology remains an active area of study. Previous studies have shown that astrocytic GJ channels and hemichannel formed by Cx43 can exert both protective and deleterious effects on RGC function in response to hypoxic or glaucomatous injury.^18^^,^^27^^,^^30^^,^^35^ In this study, we demonstrate that the expression and function of Cx43 are increased in an experimental mouse model of ocular hypertension that has been used to study mechanisms of RGC injury and neuroprotection. In addition, we show that deletion of Cx43 reduced Brn3a^+^ RGC loss, as well as mitigated damage to the inner retina and reduced Müller cell gliosis. Our data indicate that pharmacologic blockade of Cx43 channels is also beneficial and preserves Brn3a^+^ RGCs after retinal I/R injury. Finally, Cx43 deletion in astrocytes did not produce overt changes in RGC function in the absence of injury, suggesting that targeting Cx43 for neuroprotection is unlikely to affect visual transmission.

The role of Cx43 channels in retinal ischemia has been previously examined in the rat I/R model.^27^^,^^35^ Connexin-mimetic peptides, which inhibit both GJ channels and hemichannels,^23^ were shown to curtail vascular leakage and reduce neuronal loss.^27^ These effects were attributed to an increase in expression of Cx43 in endothelial cells, followed by the opening of Cx43 hemichannels in vascular cells. Although the authors did not examine whether Cx43 hemichannels are activated in the intact retina following I/R injury, primary endothelial cells in culture subjected to ischemia showed enhanced dye uptake, suggesting that hemichannels might be activated in these cells.^27^ Retinal ischemia also caused an upregulation of Cx43 in astrocytes, but the significance of this increase to vascular leakage or ganglion cell death was not explored.^27^ Our results establish that astrocytic Cx43 contributes significantly to RGC loss seen in this injury model.

Both an increase of Cx43 hemichannel activity and GJ coupling in astrocytes have been well documented in a variety of central nervous system (CNS) injury models.^49^^–^^54^ Cx43 hemichannels are normally closed in physiologic conditions and open in response to pathologic stimuli such as ischemia, oxidative stress, and glutamate toxicity. However, in retinal I/R injury, astrocytes did not show connexin-specific EtBr uptake. In contrast, we found a significant increase in GJ coupling soon after initiation of injury, supporting the notion that GJ coupling rather than hemichannel activity promotes the detrimental effect on neuronal survival. This finding is in line with other studies in the brain and in the retina that indicate that an increase in astrocytic GJ coupling expands the scope of the focal injury and exacerbates neuronal loss.^54^^–^^57^ However, we do not exclude the possibility of the contribution of Cx43 hemichannels in RGC death seen in other retinal diseases, including glaucoma and diabetic retinopathy.

Previous studies suggest that Cx43 is expressed not only in astrocytes and endothelial cells but also in Müller glia.^27^^,^^29^^,^^35^ Unlike these reports, we did not detect expression of Cx43 in Müller cells and microglia before or after ischemic injury. In addition, we did not detect dye coupling between adjacent Müller cells before or after injury. Previous studies have similarly shown that the magnitude of coupling in Müller glia, as assessed by the spread of low molecular weight tracers, is very low.^19^^,^^44^ Recent single-cell RNA sequencing studies also indicate that *GJA1*, the gene that encodes for Cx43, is predominantly found in astrocytes and endothelial cells, whereas Müller cell expression was low and restricted to a few cell types.^58^ Whether this discrepancy is due to species differences (mouse versus rabbit) is unclear and warrants additional studies.

Astrocytes respond to CNS injury by adopting a reactive state, termed *astrogliosis*, characterized by a variety of morphologic and molecular changes. Along with astrocyte reactivity, upregulation of Cx43 in astrocytes also has been observed in various models of injury in the brain.^35^^,^^59^^–^^63^ Similar to those reports, we observed that astrocytes undergo significant morphologic changes concomitant with a steady increase in expression of Cx43 protein in response to retinal I/R injury, which accompanied the loss of neuronal cells.

The mechanism underlying the deleterious effect of Cx43 on RGC loss remains to be determined. Astrocytes adopt a multitude of reactive states during the course of neurodegenerative diseases.^8^^,^^10^^,^^11^^,^^13^^,^^64^^–^^67^ These context-specific effects are governed by the action of several signaling pathways and through crosstalk with other cells, including microglia.^10^^,^^66^^,^^68^ Furthermore, the overall impact of reactive astrocytes on neuronal function varies with disease severity and duration and can lead to beneficial or deleterious clinical outcomes.^16^^,^^64^^,^^69^^,^^70^ For example, astrocyte reactivity in early disease stages mediates neuroprotective effects in glaucoma and other CNS diseases.^11^^,^^15^^,^^71^^–^^73^ In contrast, reactive astrocytes in chronic injury settings appear to actively promote inflammation and contribute to disease pathogenesis.^15^^,^^16^^,^^74^^–^^78^ In this context, gene profiling studies indicate that a subset of reactive astrocytes adopts a phenotype that is detrimental to neuronal function in glaucomatous injury and optic nerve crush.^64^^,^^79^ This transcriptionally defined subset of astroglial cells was induced in response to factors secreted by activated microglia (IL1a, TNFα, and C1q).^64^^,^^79^ Importantly, inhibiting the formation of this reactive astrocyte subset markedly increased RGC survival in glaucomatous injury.^14^^,^^64^^,^^79^ In line with these findings, selective ablation of NF-κB, a transcription factor that controls many aspects of the astrocyte proinflammatory responses, or of TNFα signaling increased RGC viability and decreased levels of proinflammatory cytokines.^75^^,^^77^^,^^78^ We hypothesize that Cx43 channels help propagate the inflammatory insult in I/R injury, transforming more astrocytes to a reactive subtype that is harmful to RGC function. Genetic ablation of Cx43 is expected to markedly reduce such propagated signaling between astrocytes and reduce RGC loss in I/R injury. Future studies will be aimed at analyzing the gene profiles in WT and Cx43 KO astrocytes after injury.

A limitation of our study is that RGC loss was measured using Brn3a labeling. Although Brn3a labels 85% to 90% of RGCs, there is evidence that Brn3a expression is reduced in certain retinal injuries, prior to RGC loss.^36^^,^^80^ Therefore, it is possible we overestimated the death of RGCs in our analyses. Future studies using different RGCs markers combined with functional studies might provide more accurate estimates of RGC loss.

## References

[1] Sofroniew MV, Vinters HV. Astrocytes: biology and pathology. *Acta Neuropathol.* 2010; 119: 7–35. 20012068 10.1007/s00401-009-0619-8 PMC2799634

[2] Vecino E, Rodriguez FD, Ruzafa N, Pereiro X, Sharma SC. Glia-neuron interactions in the mammalian retina. *Prog Retin Eye Res*. 2016; 51: 1–40. 26113209 10.1016/j.preteyeres.2015.06.003

[3] Lye-Barthel M, Sun D, Jakobs TC. Morphology of astrocytes in a glaucomatous optic nerve. *Invest Ophthalmol Vis Sci*. 2013; 54: 909–917. 23322566 10.1167/iovs.12-10109 PMC3564449

[4] Davis CH, Kim KY, Bushong EA, et al. Transcellular degradation of axonal mitochondria. *Proc Natl Acad Sci USA*. 2014; 111: 9633–9638. 24979790 10.1073/pnas.1404651111 PMC4084443

[5] Hernandez MR, Miao H, Lukas T. Astrocytes in glaucomatous optic neuropathy. *Prog Brain Res*. 2008; 173: 353–373. 18929121 10.1016/S0079-6123(08)01125-4

[6] Calkins DJ. Adaptive responses to neurodegenerative stress in glaucoma. *Prog Retin Eye Res*. 2021; 84: 100953. 33640464 10.1016/j.preteyeres.2021.100953 PMC8384979

[7] Alqawlaq S, Flanagan JG, Sivak JM. All roads lead to glaucoma: induced retinal injury cascades contribute to a common neurodegenerative outcome. *Exp Eye Res*. 2019; 183: 88–97. 30447198 10.1016/j.exer.2018.11.005

[8] Escartin C, Galea E, Lakatos A, et al. Reactive astrocyte nomenclature, definitions, and future directions. *Nat Neurosci*. 2021; 24: 312–325. 33589835 10.1038/s41593-020-00783-4 PMC8007081

[9] Sun D, Jakobs TC. Structural remodeling of astrocytes in the injured CNS. *Neuroscientist*. 2012; 18: 567–588. 21982954 10.1177/1073858411423441 PMC3713769

[10] Sofroniew MV. Astrocyte reactivity: subtypes, states, and functions in CNS innate immunity. *Trends Immunol*. 2020; 41: 758–770. 32819810 10.1016/j.it.2020.07.004 PMC7484257

[11] Sun D, Moore S, Jakobs TC. Optic nerve astrocyte reactivity protects function in experimental glaucoma and other nerve injuries. *J Exp Med*. 2017; 214: 1411–1430. 28416649 10.1084/jem.20160412 PMC5413323

[12] Wang R, Seifert P, Jakobs TC. Astrocytes in the optic nerve head of glaucomatous mice display a characteristic reactive phenotype. *Invest Ophthalmol Vis Sci*. 2017; 58: 924–932. 28170536 10.1167/iovs.16-20571 PMC5300248

[13] Anderson MA, Burda JE, Ren Y, et al. Astrocyte scar formation aids central nervous system axon regeneration. *Nature*. 2016; 532: 195–200. 27027288 10.1038/nature17623 PMC5243141

[14] Sterling JK, Adetunji MO, Guttha S, et al. GLP-1 receptor agonist NLY01 reduces retinal inflammation and neuron death secondary to ocular hypertension. *Cell Rep*. 2020; 33: 108271. 33147455 10.1016/j.celrep.2020.108271 PMC7660987

[15] Livne-Bar I, Wei J, Liu HH, et al. Astrocyte-derived lipoxins A4 and B4 promote neuroprotection from acute and chronic injury. *J Clin Invest*. 2017; 127: 4403–4414. 29106385 10.1172/JCI77398 PMC5707141

[16] Wheeler MA, Clark IC, Tjon EC, et al. MAFG-driven astrocytes promote CNS inflammation. *Nature*. 2020; 578: 593–599. 32051591 10.1038/s41586-020-1999-0 PMC8049843

[17] Theis M, Giaume C. Connexin-based intercellular communication and astrocyte heterogeneity. *Brain Res*. 2012; 1487: 88–98. 22789907 10.1016/j.brainres.2012.06.045

[18] Slavi N, Toychiev AH, Kosmidis S, et al. Suppression of connexin 43 phosphorylation promotes astrocyte survival and vascular regeneration in proliferative retinopathy. *Proc Natl Acad Sci USA*. 2018; 115: E5934–E5943. 29891713 10.1073/pnas.1803907115 PMC6042062

[19] Ceelen PW, Lockridge A, Newman EA. Electrical coupling between glial cells in the rat retina. *Glia*. 2001; 35: 1–13. 11424187 10.1002/glia.1065 PMC2479788

[20] Newman EA, Zahs KR. Modulation of neuronal activity by glial cells in the retina. *J Neurosci*. 1998; 18: 4022–4028. 9592083 10.1523/JNEUROSCI.18-11-04022.1998 PMC2904245

[21] Kang J, Kang N, Lovatt D, et al. Connexin 43 hemichannels are permeable to ATP. *J Neurosci*. 2008; 28: 4702–4711. 18448647 10.1523/JNEUROSCI.5048-07.2008 PMC3638995

[22] Orellana JA, Diaz E, Schalper KA, Vargas AA, Bennett MV, Saez JC. Cation permeation through connexin 43 hemichannels is cooperative, competitive and saturable with parameters depending on the permeant species. *Biochem Biophys Res Commun*. 2011; 409: 603–609. 21600880 10.1016/j.bbrc.2011.05.031 PMC3118918

[23] O'Carroll SJ, Alkadhi M, Nicholson LF, Green CR. Connexin 43 mimetic peptides reduce swelling, astrogliosis, and neuronal cell death after spinal cord injury. *Cell Commun Adhes*. 2008; 15: 27–42. 18649176 10.1080/15419060802014164

[24] Contreras JE, Sanchez HA, Eugenin EA, et al. Metabolic inhibition induces opening of unapposed connexin 43 gap junction hemichannels and reduces gap junctional communication in cortical astrocytes in culture. *Proc Natl Acad Sci USA*. 2002; 99: 495–500. 11756680 10.1073/pnas.012589799 PMC117588

[25] Bennett MV, Garre JM, Orellana JA, Bukauskas FF, Nedergaard M, Saez JC. Connexin and pannexin hemichannels in inflammatory responses of glia and neurons. *Brain Res*. 2012; 1487: 3–15. 22975435 10.1016/j.brainres.2012.08.042 PMC3627726

[26] Zahs KR, Kofuji P, Meier C, Dermietzel R. Connexin immunoreactivity in glial cells of the rat retina. *J Comp Neurol*. 2003; 455: 531–546. 12508325 10.1002/cne.10524

[27] Danesh-Meyer HV, Kerr NM, Zhang J, et al. Connexin43 mimetic peptide reduces vascular leak and retinal ganglion cell death following retinal ischaemia. *Brain*. 2012; 135: 506–520. 22345088 10.1093/brain/awr338

[28] Muto T, Tien T, Kim D, Sarthy VP, Roy S. High glucose alters Cx43 expression and gap junction intercellular communication in retinal Muller cells: promotes Muller cell and pericyte apoptosis. *Invest Ophthalmol Vis Sci*. 2014; 55: 4327–4337. 24938518 10.1167/iovs.14-14606 PMC4098057

[29] Danesh-Meyer HV, Zhang J, Acosta ML, Rupenthal ID, Green CR. Connexin43 in retinal injury and disease. *Prog Retin Eye Res*. 2016; 51: 41–68. 26432657 10.1016/j.preteyeres.2015.09.004

[30] Cooper ML, Pasini S, Lambert WS, et al. Redistribution of metabolic resources through astrocyte networks mitigates neurodegenerative stress. *Proc Natl Acad Sci USA*. 2020; 117: 18810–18821. 32690710 10.1073/pnas.2009425117 PMC7414143

[31] Sato T, Haimovici R, Kao R, Li AF, Roy S. Downregulation of connexin 43 expression by high glucose reduces gap junction activity in microvascular endothelial cells. *Diabetes*. 2002; 51: 1565–1571. 11978657 10.2337/diabetes.51.5.1565

[32] Hartsock MJ, Cho H, Wu L, Chen WJ, Gong J, Duh EJ. A mouse model of retinal ischemia-reperfusion injury through elevation of intraocular pressure. *J Vis Exp*. 2016; 113: 54065. 10.3791/54065 PMC5091361 27501124

[33] Osborne NN, Casson RJ, Wood JP, Chidlow G, Graham M, Melena J. Retinal ischemia: mechanisms of damage and potential therapeutic strategies. *Prog Retin Eye Res*. 2004; 23: 91–147. 14766318 10.1016/j.preteyeres.2003.12.001

[34] Akopian A, Atlasz T, Pan F, et al. Gap junction-mediated death of retinal neurons is connexin and insult specific: a potential target for neuroprotection. *J Neurosci*. 2014; 34: 10582–10591. 25100592 10.1523/JNEUROSCI.1912-14.2014 PMC4200109

[35] Kerr NM, Johnson CS, Zhang J, Eady EK, Green CR, HV Danesh-Meyer. High pressure-induced retinal ischaemia reperfusion causes upregulation of gap junction protein connexin43 prior to retinal ganglion cell loss. *Exp Neurol*. 2012; 234: 144–152. 22226601 10.1016/j.expneurol.2011.12.027

[36] Nadal-Nicolas FM, Jimenez-Lopez M, Sobrado-Calvo P, et al. Brn3a as a marker of retinal ganglion cells: qualitative and quantitative time course studies in naive and optic nerve-injured retinas. *Invest Ophthalmol Vis Sci*. 2009; 50: 3860–3868. 19264888 10.1167/iovs.08-3267

[37] Sun W, Cornwell A, Li J, et al. SOX9 is an astrocyte-specific nuclear marker in the adult brain outside the neurogenic regions. *J Neurosci*. 2017; 37: 4493–4507. 28336567 10.1523/JNEUROSCI.3199-16.2017 PMC5413187

[38] Ivanova E, Kovacs-Oller T, Sagdullaev BT. Domain-specific distribution of gap junctions defines cellular coupling to establish a vascular relay in the retina. *J Comp Neurol*. 2019; 527: 2675–2693. 30950036 10.1002/cne.24699 PMC6721971

[39] Musil LS, Goodenough DA. Biochemical analysis of connexin43 intracellular transport, phosphorylation, and assembly into gap junctional plaques. *J Cell Biol*. 1991; 115: 1357–1374. 1659577 10.1083/jcb.115.5.1357 PMC2289231

[40] Musil LS, Cunningham BA, Edelman GM, Goodenough DA. Differential phosphorylation of the gap junction protein connexin43 in junctional communication-competent and -deficient cell lines. *J Cell Biol*. 1990; 111: 2077–2088. 2172261 10.1083/jcb.111.5.2077 PMC2116332

[41] Solan JL, Lampe PD. Connexin43 phosphorylation: structural changes and biological effects. *Biochem J*. 2009; 419: 261–272. 19309313 10.1042/BJ20082319 PMC2669545

[42] Zahs KR, Ceelen PW. Gap junctional coupling and connexin immunoreactivity in rabbit retinal glia. *Vis Neurosci*. 2006; 23: 1–10. 16597346 10.1017/S0952523806231018

[43] Ball AK, McReynolds JS. Localization of gap junctions and tracer coupling in retinal Muller cells. *J Comp Neurol*. 1998; 393: 48–57. 9520100 10.1002/(sici)1096-9861(19980330)393:1<48::aid-cne5>3.0.co;2-q

[44] Zahs KR, Newman EA. Asymmetric gap junctional coupling between glial cells in the rat retina. *Glia*. 1997; 20: 10–22. 9145301

[45] Contreras JE, Sanchez HA, Veliz LP, Bukauskas FF, Bennett MV, Saez JC. Role of connexin-based gap junction channels and hemichannels in ischemia-induced cell death in nervous tissue. *Brain Res Brain Res Rev*. 2004; 47: 290–303. 15572178 10.1016/j.brainresrev.2004.08.002 PMC3651737

[46] Orellana JA, Froger N, Ezan P, et al. ATP and glutamate released via astroglial connexin 43 hemichannels mediate neuronal death through activation of pannexin 1 hemichannels. *J Neurochem*. 2011; 118: 826–840. 21294731 10.1111/j.1471-4159.2011.07210.x PMC3108012

[47] Giaume C, Leybaert L, Naus CC, Saez JC. Connexin and pannexin hemichannels in brain glial cells: properties, pharmacology, and roles. *Front Pharmacol*. 2013; 4: 88. 23882216 10.3389/fphar.2013.00088 PMC3713369

[48] Silverman SM, Kim BJ, Howell GR, et al. C1q propagates microglial activation and neurodegeneration in the visual axis following retinal ischemia/reperfusion injury. *Mol Neurodegeneration*. 2016; 11: 24. 10.1186/s13024-016-0089-0 PMC4806521 27008854

[49] Chen B, Yang L, Chen J, et al. Inhibition of connexin43 hemichannels with Gap19 protects cerebral ischemia/reperfusion injury via the JAK2/STAT3 pathway in mice. *Brain Res Bull*. 2019; 146: 124–135. 30593877 10.1016/j.brainresbull.2018.12.009

[50] Freitas-Andrade M, Wang N, Bechberger JF, et al. Targeting MAPK phosphorylation of connexin43 provides neuroprotection in stroke. *J Exp Med*. 2019; 216: 916–935. 30872361 10.1084/jem.20171452 PMC6446879

[51] Ma D, Feng L, Cheng Y, et al. Astrocytic gap junction inhibition by carbenoxolone enhances the protective effects of ischemic preconditioning following cerebral ischemia. *J Neuroinflammation*. 2018; 15: 198. 29976213 10.1186/s12974-018-1230-5 PMC6034345

[52] Frantseva MV, Kokarovtseva L, Naus CG, Carlen PL, MacFabe D, Perez Velazquez JL. Specific gap junctions enhance the neuronal vulnerability to brain traumatic injury. *J Neurosci*. 2002; 22: 644–653. 11826094 10.1523/JNEUROSCI.22-03-00644.2002 PMC6758478

[53] Rami A, Volkmann T, Winckler J. Effective reduction of neuronal death by inhibiting gap junctional intercellular communication in a rodent model of global transient cerebral ischemia. *Exp Neurol*. 2001; 170: 297–304. 11476596 10.1006/exnr.2001.7712

[54] Lin JH, Weigel H, Cotrina ML, et al. Gap-junction-mediated propagation and amplification of cell injury. *Nat Neurosci*. 1998; 1: 494–500. 10196547 10.1038/2210

[55] Cotrina ML, Lin JH, Alves-Rodrigues A, et al. Connexins regulate calcium signaling by controlling ATP release. *Proc Natl Acad Sci USA*. 1998; 95: 15735–15740. 9861039 10.1073/pnas.95.26.15735 PMC28113

[56] Xie M, Yi C, Luo X, et al. Glial gap junctional communication involvement in hippocampal damage after middle cerebral artery occlusion. *Ann Neurol*. 2011; 70: 121–132. 21786302 10.1002/ana.22386

[57] Xu G, Wang W, Kimelberg HK, Zhou M. Electrical coupling of astrocytes in rat hippocampal slices under physiological and simulated ischemic conditions. *Glia*. 2010; 58: 481–493. 19795502 10.1002/glia.20939

[58] Hoang T, Wang J, Boyd P, et al. Gene regulatory networks controlling vertebrate retinal regeneration. *Science*. 2020; 370: eabb8598. 33004674 10.1126/science.abb8598 PMC7899183

[59] Mayorquin LC, Rodriguez AV, Sutachan JJ, Albarracin SL. Connexin-mediated functional and metabolic coupling between astrocytes and neurons. *Front Mol Neurosci*. 2018; 11: 118. 29695954 10.3389/fnmol.2018.00118 PMC5905222

[60] Tonkin RS, Mao Y, O'Carroll SJ, et al. Gap junction proteins and their role in spinal cord injury. *Front Mol Neurosci*. 2014; 7: 102. 25610368 10.3389/fnmol.2014.00102 PMC4285056

[61] Nakase T, Maeda T, Yoshida Y, Nagata K. Ischemia alters the expression of connexins in the aged human brain. *J Biomed Biotechnol*. 2009; 2009: 147946. 19794823 10.1155/2009/147946 PMC2753779

[62] Nakase T, Yoshida Y, Nagata K. Enhanced connexin 43 immunoreactivity in penumbral areas in the human brain following ischemia. *Glia*. 2006; 54: 369–375. 16886200 10.1002/glia.20399

[63] Theriault E, Frankenstein UN, Hertzberg EL, Nagy JI. Connexin43 and astrocytic gap junctions in the rat spinal cord after acute compression injury. *J Comp Neurol*. 1997; 382: 199–214. 9183689

[64] Liddelow SA, Guttenplan KA, Clarke LE, et al. Neurotoxic reactive astrocytes are induced by activated microglia. *Nature*. 2017; 541: 481–487. 28099414 10.1038/nature21029 PMC5404890

[65] Khakh BS, Sofroniew MV. Diversity of astrocyte functions and phenotypes in neural circuits. *Nat Neurosci*. 2015; 18: 942–952. 26108722 10.1038/nn.4043 PMC5258184

[66] Linnerbauer M, Wheeler MA, Quintana FJ. Astrocyte crosstalk in CNS inflammation. *Neuron*. 2020; 108: 608–622. 32898475 10.1016/j.neuron.2020.08.012 PMC7704785

[67] Zamanian JL, Xu L, Foo LC, et al. Genomic analysis of reactive astrogliosis. *J Neurosci*. 2012; 32: 6391–6410. 22553043 10.1523/JNEUROSCI.6221-11.2012 PMC3480225

[68] Liddelow SA, Barres BA. Reactive astrocytes: production, function, and therapeutic potential. *Immunity*. 2017; 46: 957–967. 28636962 10.1016/j.immuni.2017.06.006

[69] Bayraktar OA, Bartels T, Holmqvist S, et al. Astrocyte layers in the mammalian cerebral cortex revealed by a single-cell in situ transcriptomic map. *Nat Neurosci*. 2020; 23: 500–509. 32203496 10.1038/s41593-020-0602-1 PMC7116562

[70] John Lin CC, Yu K, Hatcher A, et al. Identification of diverse astrocyte populations and their malignant analogs. *Nat Neurosci*. 2017; 20: 396–405. 28166219 10.1038/nn.4493 PMC5824716

[71] Mayo L, Trauger SA, Blain M, et al. Regulation of astrocyte activation by glycolipids drives chronic CNS inflammation. *Nat Med*. 2014; 20: 1147–1156. 25216636 10.1038/nm.3681 PMC4255949

[72] Okada S, Nakamura M, Katoh H, et al. Conditional ablation of Stat3 or Socs3 discloses a dual role for reactive astrocytes after spinal cord injury. *Nat Med*. 2006; 12: 829–834. 16783372 10.1038/nm1425

[73] Ben Haim L, Rowitch DH. Functional diversity of astrocytes in neural circuit regulation. *Nat Rev Neurosci*. 2017; 18: 31–41. 27904142 10.1038/nrn.2016.159

[74] Rothhammer V, Quintana FJ. The aryl hydrocarbon receptor: an environmental sensor integrating immune responses in health and disease. *Nat Rev Immunol*. 2019; 19: 184–197. 30718831 10.1038/s41577-019-0125-8

[75] Tezel G. TNF-alpha signaling in glaucomatous neurodegeneration. *Prog Brain Res*. 2008; 173: 409–421. 18929124 10.1016/S0079-6123(08)01128-X PMC3150483

[76] Brambilla R, Bracchi-Ricard V, Hu WH, et al. Inhibition of astroglial nuclear factor kappaB reduces inflammation and improves functional recovery after spinal cord injury. *J Exp Med*. 2005; 202: 145–156. 15998793 10.1084/jem.20041918 PMC2212896

[77] Yang X, Zeng Q, Baris M, Tezel G. Transgenic inhibition of astroglial NF-kappaB restrains the neuroinflammatory and neurodegenerative outcomes of experimental mouse glaucoma. *J Neuroinflammation*. 2020; 17: 252. 32859212 10.1186/s12974-020-01930-1 PMC7456390

[78] Dvoriantchikova G, Barakat D, Brambilla R, et al. Inactivation of astroglial NF-kappa B promotes survival of retinal neurons following ischemic injury. *Eur J Neurosci*. 2009; 30: 175–185. 19614983 10.1111/j.1460-9568.2009.06814.x PMC2778328

[79] Guttenplan KA, Stafford BK, El-Danaf RN, et al. Neurotoxic reactive astrocytes drive neuronal death after retinal injury. *Cell Reports*. 2020; 31: 107776. 32579912 10.1016/j.celrep.2020.107776 PMC8091906

[80] Buckingham BP, Inman DM, Lambert W, et al. Progressive ganglion cell degeneration precedes neuronal loss in a mouse model of glaucoma. *J Neurosci*. 2008; 28: 2735–2744. 18337403 10.1523/JNEUROSCI.4443-07.2008 PMC6670674

